# Stigma-driven bypassing of ART services in Northern Ghana: a qualitative case study

**DOI:** 10.1017/S1463423625100789

**Published:** 2026-01-28

**Authors:** Sadat Zakari Abugbila, Joshua Sumankuuro, Maximillian Kolbe Domapielle

**Affiliations:** 1 Department of Governance and Development Management, Faculty of Public Policy and Governance, University of Business and Integrated Development Studies, Wa, Ghana; 2 Department of Public Policy and Management, Faculty of Public Policy and Governance, University of Business and Integrated Development Studies, Wa, Ghana; 3 School of Nursing, Paramedicine and Health Care Sciences, Faculty of Science and Health, Charles Sturt University, Bathurst, NSW, Australia; 4 The West African Center for Sustainable Rural Transformation (WAC-SRT), University of Business and Integrated Development Studies (SDD-UBIDS), Wa, Ghana; 5 Centre for Migration and Security Studies, Faculty of Public Policy and Management, University of Business and Integrated Development Studies, Wa, Ghana

**Keywords:** antiretroviral therapy facilities, discrimination, Ghana, HIV/AIDS, stigma

## Abstract

**Background:**

This study analyses the relationship between fear of stigma and bypassing primary ART facilities by ART clients in the Upper East Region of Ghana.

**Methodology:**

Methodology: The study employed an exploratory case study design, involving 52 participants of: ART clients (n = 37), nurses (n = 7), a counsellor (n = 1), cadres (n = 2), pharmacists (n = 2) and data managers (n = 3) through convenient and purposive sampling techniques. Data was collected using semi-structured interview guides and analysed using a thematic framework.

**Results:**

The study provides ample evidence of the occurrence of stigma-driven bypassing of primary ART facilities by clients. The analysis shows entrenched cultural norms and values and the population’s low awareness of the efficacy of ART fuel the processes of stigma and discrimination towards ART clients.

**Strengths and limitations:**

We acknowledge the following limitations and strengths: convenient and purposive sampling procedures may not represent the views of all ART clients on bypassing primary facilities. Sensitive nature of HIV and the location of ART centres, coupled with time constraints in probing into all ART bypassing issues. Yet, given the depth of the issues presented and the scope of participants and ART facilities, we believe relevant data was generated to address the research question.

**Conclusion:**

An integrated approach could be used to address the drivers of stigma and discrimination focusing on awareness creation to undo the entrenched negative cultural beliefs around HIV transmission, and implement anti-HIV stigma legislation to eliminate prejudice towards PLHIV.

## Introduction

Adherence to antiretroviral therapy (ART) is key to achieving the United Nations’ Joint Programme on HIV/AIDs (UNAIDS) 95-95-95 HIV testing, treatment and viral suppression targets by 2025 (Stover *et al*., 2021). In 2023, the World Health Organization’s (WHO) global data on the uptake of the ART show that 86 percent of people living with HIV knew their status, 77 percent of them were receiving ART, and 72 percent were virally suppressed. In Africa, 90 percent of the estimated 26 million people living with HIV knew their status, 82 percent were receiving treatment, and 76 percent had suppressed viral loads (WHO, 2024). Evidence suggests there has been progress, although significant shortfalls persist in testing, treatment and viral suppression. The statistics have shown that 1.3 million new HIV infections and 630 thousand HIV-related deaths occur annually, thereby heightening concerns about achievability of the UNAIDS treatment targets for 2025 (UNAIDS, 2025a). In addition, these challenges threaten the objectives of the Global AIDS strategy (2026–2031), aimed at eradicating AIDS as a public health threat by 2030 (UNAIDS, 2025b).

ART has become a conventional treatment known as ‘highly active antiretroviral therapy’ or HAART. HAART is a treatment regimen involving a combination of three or more antiretroviral drugs used to manage HIV (Saka *et al*., 2023; Gandhi *et al*., 2025). HAART aims to suppress HIV replication, reducing viral load and maintaining a healthy immune system. It prevents opportunistic infections and HIV transmission risk, helping people with HIV live longer and healthier lives, although it is not a cure (Saka *et al*., 2023). Notably in Ghana and similar countries, HAART has suppressed the viral load in PLHIV, reduced AIDS-related mortalities, lowered risk of transmission to others, and has had a positive impact on the overall health of the population affected by HIV/AIDS (Saka *et al*., 2023). ART was initially piloted in Ghana for the general population and prevention of mother-to-child transmission (PMTC) in 2002, and subsequently expanded to cover treatment and counselling services nationwide in 2005 (NACP, 2015). Despite some progress in the ART coverage of the people living with HIV, the country only managed to reach 63-95-73 out of the UNAIDS 90-90-90 targets at the close of 2020, and its progress towards project 95-95-95 by end of 2025 hangs in a balance (NACP, 2020).

Previous studies have identified bypassing primary ART sites by clients as one of the key barriers to achieving project 95-95-95 in many sub-Saharan African countries (Yao and Agadjanian, 2018; Adjetey *et al.*, 2019; Domapielle *et al.*, 2024). *Bypassing* occurs when patients knowingly visit a health facility other than the one nearest to their place of residence. Several reasons often account for PLHIV bypassing including avoiding poor administration, unsatisfactory care, unethical behaviours of providers, and negative stereotypes (Ayiigah *et al.*, 2024; Domapielle *et al*., 2024). A critical factor associated with the phenomenon is fear of stigma (Masango-Makgobela *et al.*, 2013; Mee *et al*., 2020; Fonner *et al.,* 2021; Adewole *et al.*, 2022; Ayiigah *et al*., 2024; Domapielle *et al.*, 2024). Etymologically, ‘stigma’ was derived from a Greek verb ‘stizo’, which means ‘to puncture’, ‘to prick’ or ‘to mark’. Thus, these terms were expanded in Christianity as bodily marks that resemble those of the crucifixion of Jesus Christ. These stood for God’s favour. However, others conceive stigma to mean marks of disgrace, discredit, or infamy. Today, the term ‘stigma’ is applied more to social disgrace than to any bodily marks or divine favour (Link and Phelan, 2001). Importantly, stigma is conceived as an attribute that is discrediting and may deprive a person of their human dignity (Goffman, 1963). Two major of forms of stigma have been described in the literature: internal or perceived stigma, and external or enacted stigma (Mumin *et al.*, 2018; Armstrong-Mensah *et al.*, 2023). Mumin *et al*. (2018) notes that that ‘enacted’ occurs in the form of discrimination, marginalization, human rights violations, withdrawal of social support and humiliation targeted at PLHIV. On the other hand, ‘perceived’ stigma occurs when ART clients believes that they were being judged negatively or feel guilty of being discriminated against by others due to their HIV status. These thoughts and perceptions created some fear of mistreatment, which has led to psychological distress, shame, and unwillingness to disclose their positive status to others, even including their spouses. Stigma, regardless of the form, affect PLHIV’s access to ART services and other healthcare services, thereby affecting their overall quality of life (Mumin *et al*., 2018; Ayiigah *et al*., 2024).

Consistent with these conceptualizations of stigma, recent empirical studies have provided insights into the factors that drive HIV-related stigma (Parker *et al*., 2020; Şamar *et al.*, 2023). Some have argued that the key drivers of the phenomenon are socio-cultural in nature and referenced instances where study participants associated HIV infection with prostitution, promiscuity and drug abuse, and men having sex with men (MSM) (Khumsaen and Stephenson, 2017; Parker *et al*., 2020). Others have attributed the prevalence of stigma and discrimination towards PLHIV to limited awareness among the population of efficacy of the ART (Ayiigah *et al*., 2024; Domapielle *et al*., 2024). These studies have reported that the inadequate awareness of the advances in treatment development and the efficacy of ART partly accounts for their unwillingness to develop accepting attitudes towards PLHIV. The third driver of stigma is the lack of a strong enabling environment for the implementation of anti-HIV stigma legislation (Ayiigah *et al*., 2024). Although the Ghana AIDS Commission’s Act 2016 (Act 938) criminalizes HIV-related stigma and discrimination, progress has not been made in terms of implementation due to health system and political constraints (GAC, 2024): the consequence of this is the prevalence of employment-related HIV stigma both in the formal and informal work settings (Ulasi *et al*., 2009; Sangaramoorthy *et al.*, 2017; Mumin *et al*., 2018; Dewi *et al*., 2020).

Therefore, this study analyses the relationship between fear of stigma and bypassing primary ART facilities by ART clients in the Upper Eat Region of Ghana. The objective is to provide an understanding of this relationship to inform the design and implementation of interventions that would eliminate stigma as a barrier to the uptake of ART.

## Methodology

### Study setting

The study was conducted in the Upper East Region of Ghana, specifically at the Upper East Regional Hospital in the Bolgatanga Municipality, Navrongo District Hospital (War Memorial Hospital), Bongo District Hospital and the Builsa North District Hospital in Sandema. The region has a population of 1,301,226, comprising 48.5 percent male and 51.5 percent female (GSS, 2021). The majority of the population (74.4%) reside in rural communities, and less than half (48.1%) of the region’s population is literate, which falls short of the national average (69.8%) (Domapielle *et al*., 2022; Sarkpoh and Domapielle, 2022; Domapielle *et al*., 2023). Additionally, 28 percent of the total population is extremely poor, 37 percent is poor, and 35 percent is categorized as non-poor (GNHR, 2023). Consistent with the mandate of the Ghana Health Service (GHS), the region’s healthcare delivery system is hierarchical, with four levels: regional, district, sub-district, and community. The regional hospital in Bolgatanga serves as the region’s secondary referral facility. Additionally, there are eight district hospitals, 101 sub-district health facilities, 348 Community Health Planning Services (CHPS) clinics, and 24 ART centres (GHS, 2021).

Compared to the national average [63-95-73], the Upper East Region recorded a lower score of 51-78-51 (Boah *et al.*, 2023). This is unsettling considering that at the close of 2023 the region had documented a total of 5776 cases, 339 new infections, and 230 HIV-related fatalities (Boah *et al*., 2023). Moreover, HIV prevalence among the adult population in the region was 1.9 percent (GAC, 2020) with ART coverage of 77.68 percent (GAC, 2020). This is coupled with distinctive socio-cultural stereotypes related to stigma towards people living with HIV (Ayiigah *et al*., 2024). These indicators provide the basis for analysing the relationship between stigma and bypassing ART sites by clients in the region.

### Research design

The study employed an exploratory case study to understand whether fear of stigma and discrimination influences bypassing of primary ART facilities in the Upper East Region. Yin (2018) states that a case study is ‘an empirical inquiry that investigates a contemporary phenomenon within its real-life context, especially when the boundaries between the phenomenon and context are not evident’. We adopted this design to explore in-depth ART clients’ experiences of stigma and discrimination and whether the fear of being tagged with a tainted image has a relationship with bypassing their primary ART centres to seek care in more distant facilities.

### Health facility selection

The selection of the region, the districts, and the staff of ART facilities was carried out using purposive sampling. Upper East Region was selected for the study because it not only has a high HIV prevalence (1.9%) but it also has a high ART coverage in the country (77.68%) (GAC, 2021). Four health facilities were purposively selected for the study to understand the different lived experiences and factors influencing bypassing of primary ART clinics across the various levels of care and geographical locations (GSS, 2021). For example, the regional hospital is at Bolgatanga; a more urbanized area than Navrongo, Bongo, and Sandema. Bolgatanga Municipality is home to secondary and primary levels of health care in the region. The district hospitals in the Sandema, Bongo, and Navrongo are relatively under-resourced and provide mainly primary healthcare services (GHS, 2021). For the selection of the ART clinic, we considered the ART clinic that had the highest number of enrolments in each district.

### Study participants

Men and women who were receiving ART in facilities in the four selected districts participated in the study. Healthcare providers at the various ART facilities were also included in the study. All participants were within the ages of 18–60 years (Table 1).

**Table 1.** Demographic profile of the ART clients

Characteristic	Categories	Number of participants	Percentage
Location of ART facilities	Bolgatanga (RHB)	12	32.4
	Navrongo (WMH)	12	32.4
	Bongo (BDH)	8	21.6
	Sandema (SDH)	5	13.6
Gender	Male	10	27
	Female	27	73
Nature of residential location	Urban	10	27.79
	Peri-urban	6	16.10
	Rural	21	56.10
Age	18–25	13	36.62
	26–35	18	49.09
	>35	5	14.29
Religion	Christian	18	48.05
	Islam	17	45.71
	Traditional/others	2	6.23
Ethnicity	Kassena Nankana	11	29.61
	Guruni/Frafra	19	48.83
	Others	7	21.56
Current marital status	Not married	9	24.4
	Married	11	29.7
	Divorced	5	13.5
	Widow/widower	12	32.4
Education	No formal education	11	25.97
	Primary	5	15.84
	JHS	9	21.56
	SHS	6	17.66
	Tertiary	7	18.96
Occupation	Formal sector employee	6	15.32
	Vocation	9	23.12
	Trade	5	15.06
	Farming	7	20.00
	Unemployed	10	26.49
Years on medication	1–2	8	23.38
	3–4	11	27.79
	5–6	7	18.96
	7–8	6	15.32
	9+	5	14.55
Viral Load Suppression	Yes	17	47.01
	No	20	52.99

Field data collected between May 2024 and July 2024.

#### ART clients and healthcare provider sampling

We employed convenient sampling to choose ART clients. We preferred convenient sampling because ART clients do not strictly follow a schedule for visiting ART centres, making it difficult to know the clients who might visit the facility on a particular date and time; therefore, it was necessary to interview any client who visited the facility that met our selection criteria.

Healthcare providers were selected using purposive sampling procedures. We employed these sampling approaches, primarily to generate relevant data to address the research aim.

Overall, a total of 52 participants took part in the study: ART clients (*n* = 37), nurses (*n* = 7, two nurses from three clinics and one nurse from one clinic), a counsellor (*n* = 1), cadres (*n* = 2), pharmacists (*n* = 2) and data monitors/officers/managers (*n* = 3).

### Participant eligibility criteria

We considered participants who were at least 18 years of age, living with HIV/AIDS, and had been a bypasser. The Upper East Regional HIV focal person, ART nurses, ART clients, data managers and pharmacists were purposively selected because of their in-depth knowledge of bypassing.

### Design of interview guide

Semi-structured interview guides were designed and used to collect the data. The guides were designed using knowledge from the review of the literature and the extensive experience implementing HIV-related projects and conducting research on HIV/AIDS (Ayiigah *et al*., 2024; Domapielle *et al*., 2024; Manu *et al.*, 2024). To assure data credibility, the first drafts of the interview guides were pre-tested with ten ART clients in a facility not among those selected for the study. Interview questions were checked for errors, adequacy, clarity and ambiguity, and were appropriately corrected before the data collection.

### Data collection

Through the interviews with ART clients and ART clinic nurses, data was collected from ART sites; whereas the Regional HIV focal person, data managers and pharmacists were interviewed in their offices. All the interviews were conducted face-to-face with the participants. The rationale for drawing on this approach was to make it possible to generate relevant data to understand the specific factors influencing the increased occurrence of bypassing primary ART facilities by ART clients. The interviews were conducted from May to July 2024. It must be acknowledged that the content of the interview guide for both groups of participants was similar – factors influencing bypassing and adherence to ART medications. Therefore, we stopped interviewing when we realized no new information was generated after interviewing additional participants. Each interview lasted between 45 minutes and one hour. Interviews were conducted in English for participants who understood and spoke it, and in the native language for participants who preferred to speak their mother tongue. All the interviews were audio-recorded with prior information and informed consent of the participants. Prior to the field work, one author (SZA) who was a postgraduate student, received a three-day extensive refresher training on ethics in research, data collection, techniques in conducting effective interviews and data management. Two authors, MKD and JS, are experienced researchers in health services, health systems and global health, and have extensive experience conducting qualitative research. SZA is a native of the study area and therefore facilitated collaboration with the Regional/District Health Directorates and the ART clinics.

### Data analysis and presentation

Data analysis began in the field where notes were taken to support the actual analysis. Audio recordings were played back to participants to confirm or disconfirm. This was necessary to ensure accuracy of the information supplied. We adopted multiple strategies to ensure the robustness of the data. First, all audio tape-recordings were transcribed by a professional transcriber in the language of the recording and subsequently translated to English for the analysis. All authors validated the transcripts by replaying the tapes. Errors detected were corrected in the text. We held meetings to validate the transcripts and reconcile discrepancies that emerged. The analysis took a collaborative process among the authors where an online platform – MURAL, was created and used to develop a coding framework. The transcripts were then shared among authors to manually code them. This continued until all the transcription was completed. The codes were later merged into themes and selected participants lived experiences selected to support themes. Inter-coder reliability was established by discussing discrepancies in codes and appropriately resolved them. The thematic analytical framework was employed to synchronize and visualize the factors that influence the bypassing of primary ART clinics among ART clients in the study settings.

### Researchers’ positionality

During the field work, our positionality shaped every step we took throughout the process. As public health researchers, we recognized our background, our professional training and personal experiences, which positively influenced how we approached ART clients, healthcare providers, and how we interpreted their responses. Indeed, our educational background provided some framework to understand the systemic and individual factors that contribute to health-seeking behaviours of ART clients and why they bypassed primary ART clinics, especially given the complexities of trust, and the sensitive nature of HIV status. While SZA originally comes from one of the study districts, it did not influence his reasoning. In fact, we upheld international standards and ethics in research, and maintained a professional ethos to ensure the integrity of the research. Moreover, we were committed to reflexivity, and constantly questioned how our assumptions, beliefs, and affiliations may impact a participant’s decision to participate in the study. By focusing on the research aim, the findings have emerged from an ethical, respectful, voluntary participation, and relevant conclusions have been drawn to help inform policy and ART service delivery within the study area and similar settings.

### Ethical considerations

Ethics clearance was obtained [#NHRCIRB648]. Participation in the study was voluntary, participants were assured of anonymity, and their responses were treated as confidential. The participants were interviewed at only the ART clinics when they visited for their routine medication and treatment. The secluded venue was purposively chosen to prevent exposing them to public stigma or any form of harm. They were also offered the opportunity to withdraw from the study if they did not wish to continue. These assurances were contained in the consent form, which was explained to them. Those who showed interest in participating in the study signed/thumb printed a written consent form. All participants gave prior written informed consent before the interviews. Overall, the study was conducted in accordance with the Declaration of Helsinki.

## Results

**Figure 1.**
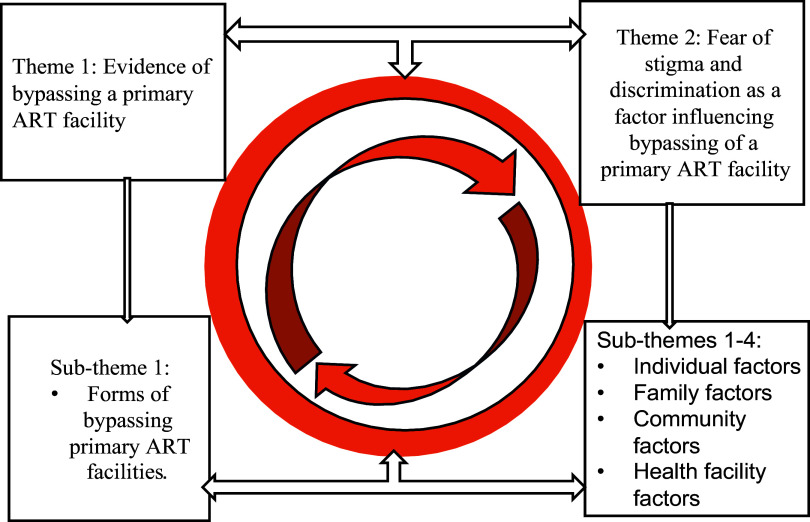
Factors influencing bypassing primary ART facilities.

Two overarching themes emerged from the data analysis: evidence of bypassing a primary ART facility; and fear of stigma and discrimination as a factor influencing bypassing of a primary ART facility. The second theme had four sub-themes: individual, family, community, and health facility factors (see Figure 1).

### Theme I: evidence of bypassing a primary ART facility

Bypassing of designated primary ART health facilities by people living with HIV was common in this study. This phenomenon was shown in the participants’ accounts of their ART medication access, and why they bypassed nearby primary ART clinics in the study area. Bypassing occurred for several reasons, and it occurred in different forms, namely bypassing by ART clients who were within the study region; PLHIV bypassing into the study region; and those who travelled from Burkina Faso to the study region to seek ART medications and other care.

#### Sub-theme 1: forms of bypassing primary ART facilities

Key informants, specifically ART nurses and data managers at the various health facilities, acknowledged a high occurrence of clients bypassing primary ART facilities for HIV/AIDS healthcare services and support. We found that clients mostly bypassed the ART facilities in Navrongo, Bongo and Sandema to access care from the ART clinic at the regional hospital. An even more worrying trend was observed where bypassing occurs both intra-regionally and inter-regionally. The intra-regional bypassing involves clients bypassing ART facilities within the study region; however, it was found that some ART clients came from other regions of Ghana. A nurse noted that:

*‘Over 80 percent of our clients are bypassers from other districts. I can state with the data we have as a primary facility in the region that up to five of the clients you are seeing here are from other districts which have ART facilities around them providing ART services’ (RDM, HD).*

An ART nurse noted that:

*‘The people who you see around, over half of them came from Wa, Tamale and even Kumasi. The woman who they (midwives) called us that she just delivered came from Tumu to deliver here because this is where she takes her medicine. There are others who have relocated to Kumasi and Techiman but have refused to take transfer to their closest facilities and still bypass to Bolgatanga for their medication including viral load testing. And so, bypassers are the most common clients we see here. And beyond those who come from Navrongo and Bawku and Bongo, there are a lot of them who come from the Sissala areas in the Upper West Region’. (Clinic in-charge, DH 1).*

Furthermore, the location of certain facilities, such as the Navrongo War Memorial Hospital near the Burkina Faso border, have been overwhelmed with international bypassers. PLHIV from Burkina Faso bypass primary ART clinics to access medication and treatment at the ART facility in that hospital. Key informants at the facility reported an increase in Burkinabe nationals accessing health services beyond ART facilities closer to them in Burkina Faso.

*‘There are ART clients who come from neighbouring Burkina Faso to access ART. This is because Burkina Faso is just few kilometres from here and so they prefer to sometimes come for their medicines here than go to their country’s facilities’ (DO, H2).*

One of the nurses noted that,

*‘Today being an ART clinic day, we have received at least five Burkinabes. They feel comfortable accessing ART here because unlike their communities in Burkina Faso where they are well known, no one knows them, so their HIV-positive status is concealed’. (Nurse In-Charge, H2).*

However, this trend is not observed in all ART facilities within the region. The most deprived facilities across the region see fewer bypassers. For example, in the Bongo District Hospital, only one bypasser was receiving treatment at the time of our visit to the facility. This can be attributed to factors such as the lack of awareness among PLHIV about the availability of ART services in the district. A client at the WMH ART facility in Navrongo had this to say about lack of awareness of the health facilities providing ART services:

*‘I travel here from Tamale for my medicine. Until last week I did not know Bongo has an ART facility else I would have gone there because nobody knows me over there. I am stuck here because I do not even know the route to Bongo’ (Male PLHIV 1, WMH).*

An ART client who bypassed his primary ART facility and sought care at the Bongo hospital ART centre stated that:

*‘Other ART facilities in the region are not known. I was diagnosed in Navrongo when I went to visit a friend and fell sick and that was when I realized I was positive to HIV. I thought the region had few ART facilities until recently, when I visited the Bongo facility. It is hidden and because it lacks other health services, I doubt people will bypass to this place’. (Male, PLHIV 2, BDH).*

### Theme 2: factors influencing bypassing of ART facilities

Participants mentioned various factors associated with bypassing of ART centres. These include the individual factors, family-related factors and community level factors. These factors in their individual or combined form can influence the bypassing decisions of ART clients.

#### Sub-theme 1: individual factors [social status and profession]

***a. Social status***

Some ART clients were community leaders, church leaders, pastors’ spouses, and popular radio presenters. For them, their social status meant that they concealed their HIV-positive status from the public, and the way to accomplish this was by bypassing their primary ART facility to access care in facilities where the secrecy of their HIV status is protected. A pastor’s wife explained why she bypasses her primary ART centre in Bolgatanga to access care at the ART centre in Sandema:

*‘The main reason I leave Bolga to come is because my husband is a pastor. Many of the doctors and nurses know me. So, if I go to Bolga and someone sees me, the news will soon spread in the town and our church members will stop coming to church. My husband is also HIV positive and because of our status in the community and in the church, it will be disastrous if anyone gets to see me around the ART facility in Bolgatanga’. (Female, PLHIV 3, SDH).*

A traditional leader with whom we interacted at the War Memorial Hospital ART centre in Navrongo had this to say:

*‘I am a traditional leader in my community. In fact, I am a sub-chief under a paramountcy and what do you think will happen if my people find out that even their leader has HIV/AIDS. I might lose my chieftaincy position. Even if I do not lose the stool, I will lose my authority and power. Imagine telling your community people to undertake an exercise and everyone is murmuring or even when there is a gathering, and everyone is murmuring because their chief is HIV positive. How would you even sit among the crowd or community leaders to take part in decision making? This is the reason why I travel all the way from Kumasi to come here to take my medication’ (Male, PLHIV 7, WMH).*

Some participants noted that a person’s popularity is often linked to their activities. Certain PLHIV are highly visible and well-known in their communities, possibly due to their family background, social status, or active involvement in social activities. Because of their prominence, these individuals often feel compelled to visit only remote ART facilities to keep their HIV status a secret. A popular person who is a male ART client made the following statement:

*‘I am not even known by face because I am a radio person. I speak to a lot of issues though many people within the region may not recognize me when they see me in town. However, because of the popularity of my name, people easily relate, and such I am afraid to visit the ART facility at the Bolgatanga Regional Hospital for my medicine’. (PLHIV 10, SDH).*

***b. Profession of the client***

It also emerged that ART clients in some professions, like nursing, medicine, and security personnel, prefer to access ART far away from their primary ART facilities for fear of stigma and discrimination. An ART client who bypassed the Bolgatanga regional hospital ART centre had this say:

*‘I will be discriminated against if colleagues get to know my status. So, I come here to keep my information away from them. There was an instance when a nurse was highly discriminated against by colleagues at the regional hospital. This issue became a big issue because after they faced ethics committee, people started the gossip and finger pointing. It is a bad thing especially coming from people you expect to know better’ (Female, PLHIV 9, WMH).*

Similarly, ART clients in the security services alluded to the possibility of being denied certain roles and even career promotions if their HIV-positive was made public. For this reason, they would bypass nearby ART facilities and travel far way to access care. A security officer who accesses his treatment at the ART facility in Bongo said this:

*‘As a security officer, I travel from Bawku and come for my medication here as and when my presence is required especially for viral load testing. Even if I have any other illness, I come here to take my medication because there is so much politics in the security services and if one finds out that this is who you are, they will use that to determine the pace of your promotion’. (Male, PLHIV 5, BDH).*

We also found that ART clients who engage in certain economic activities face a high risk of losing their livelihoods if their HIV status becomes public knowledge. To safeguard their source of livelihood, many choose to travel long distances to access healthcare services. This precaution is particularly common among those working in the food industry, where stigma associated with HIV can deter customers from patronizing their catering services. These concerns highlight the economic vulnerability faced by individuals whose occupations are at risks of stigma because of their positive HIV-status. A client said this about employment-related stigma, which necessitated bypassing her primary ART clinic:

*‘I was a food vendor in Accra with my uncle before I came here. I attended a catering school and paid expensive fees to establish myself. I started very big and had a contract almost every single day to the extent I even established a restaurant selling northern meals but look at my situation now. I lost my customers because my uncle blew the alarm about my HIV status after I confided in him. When I did not heed his requests to stop selling food and he went ahead informed people about my status. Now, everyone stopped buying my food and those referrals I used to get all were gone within a twinkle of an eye. I have started something here and now I am hiding to travel for my medication and, so, I cannot risk it again. I am not the only person. When we had a peer talk here one of the days, many of the women were victims of the same situation and so I must be wise or else I will go back to zero’ (female PLHIV 12, SDH).*

A concerning happening was related to women losing their jobs when their superiors and employers became aware their HIV-positive status. This phenomenon was predominant among domestic workers and PLHIV who engaged in informal menial jobs for their livelihood. During the interviews, it was reported by a management staff at the regional hospital that PLHIV resorted to bypassing primary healthcare facilities due to these unfortunate behaviours towards ART clients in their communities:

*‘The issue of women with PLHIV losing their works is very common. Even those who serve at eateries have suffered this and so they are serious bypassers although they earn very little, but they prefer to travel longer distances to access ART medication and treatment. Those we call ‘asiibi’, the ones who wash the bowls at ‘chop bars’ have suffered a lot and so they also bypass’ (PLC 2).*

#### Sub-theme 2: family-related factors

Some people attribute HIV/AIDS to curses against people who have offended the gods by engaging in sinful activities such as prostitution or promiscuity, and for this reason PLHIV do not deserve empathy and support. To avoid bringing shame to one’s family by being tagged as a deviant, ART clients have had to keep information about their HIV status a secret. A participant had this to say about bypassing his primary ART centre to avoid leaving the name of his family in a bad light:

*‘I do not use my primary ART clinic because sincerely I do not want my family to abandon me. My wife would not even respect me if she found out I am positive to HIV. I will be abused for the rest of my life and happiness will forever be something of the past’ (Male, PLHIV 13, BDH).*

The consequences of disclosure of status have in some instances been excessive and the fear of such treatment from relatives explains why bypassing primary ART facilities and travelling farther distances to access ART medication and treatment is common among PLHIV on ART services. A paralegal counsellor from one of the ART facilities indicated that:

*‘There was an instance when a male client came to the facility and reported that someone had informed his wife about his status and that led to the wife divorcing him. We invited the wife and counselled her, but she went ahead with the divorce’. (PLC)*

On a positive note, however, some reported that although their families were aware of their HIV status, they remained supportive, and their status had been protected over several years. This has been a means of protection for them. Bypassing was used as a route to prevent the knowledge of their HIV-positive status beyond the immediate family. A bypasser responded to a question on whether his family had been supportive:

*‘No one knows about my HIV status except my wife. So, I haven’t experienced discrimination from others. Keeping my condition private has shielded me from the potential stigma and judgment that often come with an HIV diagnosis. Because my status is a secret, I haven’t faced the negative reactions or exclusion that many people with HIV encounter from their family, friends, or community. This privacy allows me to maintain my social relationships and professional life without the fear of being treated differently or unfairly due to my health status. My wife’s support has been crucial in helping me manage my condition without the added burden of societal discrimination’. (Male. PLHIV 2, BDH).*

#### Sub-theme 3: community-related factors

PLHIV reported experiencing stigma within their communities when their status was known. This fear of stigma and related consequences influences them to bypass local ART facilities where their health status was known, to seek care at ART clinics where they were not known. Participants reported that news about their HIV status often spread quickly within the community, leading to gossip and rumours that tarnished their reputation. They experienced social exclusion, where they were no longer invited to community events or social gatherings, increasing their isolation. Some participants faced verbal abuse and mixed derogatory comments from community members, which reinforced their sense of being unwelcome and judged. A female ART client said this in response to a question on why she bypasses her primary ART facility:

*‘I take my medication in a hidden place because I do not want anyone in the community to know about my HIV status. I cannot afford that risk and so I travel here from a remote area, which takes an hour by bus, to get my medication. The stigma in our community is high and if I received treatment locally, my social life and work will be negatively affected’ (Female, PLHIV, RHB).*

Another participant noted that:

*‘People were always pointing at me and gossiping about incidences I have nothing to do with, simply because they wanted to provoke me into responding. They used any opportunity to insult me about my HIV status. Faced with this constant harassment and lack of peace, I have no option but to relocate to a new place. From there, I now travel a longer distance to access ART medication, ensuring that I can maintain my privacy and avoid the relentless discrimination and stigma I faced in my previous community’. (Female PLHIV, WMH).*

It was found that some participants bypassed primary health facilities due to fear of stigma from family and communities’ members who previous saw them at the ART clinic. While, the issue of the poor location of the ART clinic within the settings of the hospital, it also points to the level of actual stigma that some PLHIV suffer. A female PLHIV shared her thoughts in the quote:

*‘When I was diagnosed, there was this woman who saw me entering the ART clinic and quickly spread the news in the community that I was HIV-positive. It forced me to change my facility because I didn’t want further embarrassment. So, I moved to this ART clinic and when I am travelling no one can tell whether I am going to the hospital or not’. (Female PLHIV, SDH).*

#### Sub-theme 4: health-facility related factors

It emerged that one of the most common reasons attributed to bypassing primary ART facilities is the presence of family and relatives working at those primary ART facilities who PLHIV feared would not keep their status confidential. This concern is more serious if they already have strained relationships with their families or in-laws. The risk of their HIV status being disclosed by direct or distant relatives working in the hospital is a significant deterrent. This fear of being exposed, either intentionally or accidentally, leads many ART clients to seek care at alternative facilities, where they feel their privacy will be protected.

*‘The main reason why I bypass the Bolgatanga regional hospital ART centre and come to the WMH ART in Navrongo is my husband’s cousin is a nurse at the Bolgatanga regional hospital ART centre, and my fear that she would disclose my HIV-positive status to others in the community I have chosen to access ART at the WMH’. (Female, PLHIV, WMH).*

However, some participants felt reassured that they received treatment from a health provider they knew. A female PLHA noted that knowing the health worker on a personal level sometimes increased their trust in the healthcare process, as they believed the worker would be more attentive and committed to providing quality care.

*‘I have a relative working here, which makes me feel that I have a better chance of receiving quality treatment. He is the son of my elder brother, so even when I experience other health issues, he is there to help me. I am diabetic and hypertensive, and he even picks up my medications for me. This connection has made it more convenient for me to visit this facility, even though there are other clinics closer to my home’ (Female PLHIV, SDH).*

## Discussion

In this study, we found increased occurrence of ART clients bypassing their primary ART sites to access care in other ART sites not designated as their primary ART centres. We also uncovered that the fear of stigma and discrimination was significantly associated with the phenomenon of bypassing primary ART centres in the study setting. The findings are discussed in the ensuing paragraphs in relation to previous research findings.

There is compelling evidence of ART clients bypassing their primary ART sites to access care in distant ART sites. Specifically, four out of 10 ART clients who participated in the study were bypassers. Indeed, it emerged in this study that bypassing ART sites occurred in three forms, namely intra-regional bypassing, inter-regional bypassing, and international bypassing. Intra-regional bypassing involves ART clients in the region seeking ART in centres outside the district where their place of residence is located. Quite often, the preferred ART centre is further away from their place of residence than their primary ART centre. This form of bypassing was more common compared with the inter-regional and international bypassing of ART sites. We also found the prevalence of inter-regional bypassing where ART clients travelled from other regions within the country to access ART in the Upper East Region. We found cases of PLHIV who bypassed ART clinics in Ashanti, Bono East, Upper West, and Northern Regions to the Upper East Region to obtain ART services. In terms of international bypassing, it emerged that some ART clients travelled from Burkina Faso to seek care in the Upper East Region. This form of bypassing was common among clients who reside in communities near the Ghana-Burkina Faso border. However, some international bypassers travelled beyond the ART centres that were located at the border communities in Ghana to the regional hospital in Bolgatanga, which they consider provide their preferred standard of care. Whereas the discussion of bypassing of ART sites by clients in Sub-Saharan Africa is not entirely new (Ayiigah *et al*., 2024; Domapielle *et al*., 2024; Masango-Makgobela *et al*., 2013; Mee *et al*., 2020), the occurrence of intra-regional, inter-regional, and international bypassing of ART sites in this study adds a new dimension to our knowledge and understanding of the three-dimensional occurrence of the phenomenon in the study setting. This understanding will inform the design and implementation of interventions to increase access to ART services in the country. Consistent with Mumin and colleague’s conceptualization of stigma, we have described the findings along two dominant forms: perceived or internal or imagined and enacted or external stigma.

**a. Perceived stigma**

Stigma was found at the individual PLHIV level. In this study, it was found that participants with social standing, such as pastors and traditional rulers/leaders, bypassed because they perceived that their subjects and congregants would have disaffection for them should their status be known. Therefore, within the Christian community in this study, bypassing was crucial for the clergy, for example, because they were popular within their municipality and the neighbouring communities and some nurses and doctors were worshippers at the church where they were leaders. Previous studies have shown that the church groups often hinder dialogue and critical thinking about HIV because they portrayed the condition as resulting from immorality and sin, and restricted ‘open language’ about it consistent with the words in the Book of Ephesians 5:3. However, open language could help challenge stigma and encourage social support and critical thinking around the condition and its potential causes (Campbell *et al.*, 2011). In a South African study, some pastors asserted that HIV was demonic and consistent with John 10:10 (Azia *et al.*, 2025). In a complicated fashion, churches in Namibia believe and preached acceptance of PLHIV, yet held the view that HIV was contracted through sex, which left PLHIV as targets of criticism. In that study, it concluded that preachers and churches promote stigmatizing and discriminatory attitudes based on fear and prejudice, pronouncing harsh moral judgements on those infected, reducing the issues of AIDS to mere moral issues (Magura *et al.*, 2025). A recent study in Cape Town, South Africa involving 20 pastors of Pentecostal churches noted that there was poor knowledge of ART. They perceived it as being caused by witchcraft or sinful acts such as prostitution, fornication, adultery or extramarital sex and then prescribe repentance and prayers as the remedy, which affects adherence to ART (Azia *et al*., 2025). By implication, church members may have to bypass primary ART facility if they have to disobey this prescription. Yet, mixed findings were reported in Malawi and Senegal, where pastors and Muslim clerics used their influence and platforms to speak against hate and stigma towards colleagues and members who were HIV-positive (Ansari and Gaestel, 2010; Trinitapoli, 2011). Pastors bypassing primary ART facilities to avoid being known by other church members and the community was potentially because they may have preached about their spiritual power to heal HIV, which has become a widely shared sermon in many previous publications. In Southern Africa, social media and television platforms were reported to have frequently advertised the miraculous healing of HIV/AIDS, including prophecies on successful supported by Bible verses (Sharpley and Kaunda, 2020).

Similarly, we found that traditional leaders embarked on inter-regional bypassing due to similar perceived stigma and discriminatory claims. For example, a traditional ruler bypassed his primary ART clinic in the middle belt of Ghana and travelled to the Upper East Region to access ART services because of fear of losing his position and the need to maintain his respect and honour among his subjects. HIV-related stigma, which led to bypassing by traditional authorities in this study, appears to be widespread and consistent with the type of discrimination and stigma observed towards local rulers in Malawi and Eswatini (News, 2003; Kumwenda *et al*., 2023). In the Malawian context, respected community leaders faced intense gossip, castigation and stigmatization from family and community due to positive HIV-status (Kumwenda *et al*., 2023). Similarly, a Chief in Eswatini publicly disclosed his HIV-positive status and was subjected to all forms of stigma and shame, which primarily stemmed from the belief that sex with a virgin could cure AIDS (News, 2003). These two community leadership groups highlight an intersection between faith, leadership and stigma-led bypassing of primary ART facilities.

In addition, it was found that some participants bypassed primary ART clinics to conceal positive status from family members and friends, including spouses. Indeed, it was found that some HIV-positive men who lived with HIV were unwilling to disclose their status to their non-positive wives. This happened for fear of losing their marital relationships, and respect and honour from their spouses and relatives. Additionally, it was found that some cases of bypassing were related to families spreading the HIV-positive status of relations, which ultimately led to business closures and irrecoverable losses. Indeed, the findings have shown that stigmatizing behaviours were common.

We observed another intriguing reason for bypassing, being that some clients bypassed ART clinics because staff or healthcare providers in those health facilities would know about their positive status to HIV. This meant that relatives and friends with HIV could not even receive medical assistance from their relatives who were healthcare providers because of perceived or enacted stigma.

It has become a concomitant challenge in the campaigns to achieve the TREAT ALL Policy by the World Health Organization. But we observed that in countries with a high prevalence of HIV. For example, ART clients had to make special arrangements to avoid primary ART clinics in South Africa. Some travelled distances and at odd hours to avoid stigma and preserve confidentiality of their status (Kagee *et al*., 2011). On the contrary, and quite paradoxically, some PLHIV bypassed primary ART facility so they could receive care from their preferred healthcare staff/provider. This occurred in a manner that even those who go on job transfers still travel longer distances to receive their medication and other HIV healthcare. These findings affect adherence to ART and some eventually miss their appointments. However, PLHIV bypassing primary ART facilities was reported in a scoping review in sub-Saharan Africa and many other studies (Magura *et al*., 2025). In that scoping review, stigma-driven bypassing was orchestrated by gossip, breaches of confidentiality, and anticipated shame. This was found to be a significant barrier that discourages patients from seeking or adhering to ART.

**b. Enacted stigma**

Enacted stigma remains a pervasive challenge for PLHIV, which leads to social, emotional and economic harm to them. In this study, we found that stigma and discrimination from family and spouses, especially it was observed that just a few PLHIV shared their HIV-positive status with their ‘significant others’, essentially, to prevent the stigma and discrimination their colleagues had suffered. Thus, avoiding discrimination from family, work colleagues and the general society was the primary ‘push’ factor for bypassing primary ART clinics in this study. Concerning actual stigma towards PLHIV in this study, we found that the phenomenon was high in their community where those whose status was known experienced discrimination, and lost social connections. This compelled them to bypass the nearest or primary ART clinics with some embarking on long distance journeys to access ART services. Previous research has reported similar findings. For instance, a Nigerian study found that PLHIV faced rejection and maltreatment from friends, family, and spouses following disclosure of their status (Adeagbo *et al.*, 2023). Indeed, in Ghana, PLHIV were excluded from home matters and denied sharing basic home arrangements including meals and cooking utensils with other family members (Nutor *et al*., 2024). Meanwhile, PLHIV need the support of family more now than before. In the Volta Region of Ghana, it was noted that women travelled distances of over 100 km to access ART because they wanted to keep their HIV status private (Manu *et al*., 2024).

Furthermore, at the community level, some bypassers were mistreated i.e., mocked, verbally abused and suffered because of gossip due to their HIV status. To restore peace, and avoid the harassment and maintain privacy, bypassing primary ART facilities was the most prudent and immediate measure for PLHIV. There were potential challenges related to the citing of ART clinics within the hospital, with some found within the out-patient department of the health facilities. Similar finding was reported in previous research in the Upper East Region (Ayiigah *et al*., 2024). Thus, to prevent relatives and community members from seeing them, they had to bypass primary ART facilities. Janek and colleagues conducted a systematic review on men living with HIV in sub-Saharan Africa. The findings showed that PLHIV were socially avoided in public places and even at the family level. Some lost their jobs and general exclusion across their home communities was quite appalling, which led to bypassing for ART clients whose status were not known. Indeed, it encouraged non-disclosure to even family members (Janek *et al.*, 2024).

The analysis has demonstrated that Ghana AIDS Commission and other stakeholders need to renew their efforts to prevent providers from mistreating, disclosing HIV status, and providing substandard care to PLHIV. These will restore the trust to utilize ART services from primary ART facilities. Besides this, the ‘good old HIV/AIDS videos’, which were used to educate communities on HIV, its mode of transmission and the support PLHIV need, may reignite and create some awareness to dispel the stigma and discriminatory treatment towards them often due to ignorance. For example, a study in South Africa revealed that pastors who demonized HIV did not even know the meaning of the acronym AIDS (Azia *et al*., 2025).

Furthermore, the findings on both perceived and enacted stigma, which partly account for bypassing primary ART facilities, indicates that the attitude towards anyone going into a HIV clinic implies that people could no longer do voluntary walk-in to test for HIV, thereby potentially affecting early detection of infections rates. It means that the present behaviours and discriminatory tendencies could forestall the campaign to increase enrolment in ART and adherence thereof. We advocate for continued education using religious leaders, traditional rulers, teachers, and other respected persons in our communities to speak out against stigma, and model supportive behaviours. To address stigma-driven bypassing, we suggest family-based counselling, which may improve family acceptance of PLHIV. Finally, we recommend intensification of large-scale campaigns using social and electronic media to dispel the myths about HIV transmission, normalize HIV testing and treatment, and highlight the rights and dignity of PLHIV.

Finally, and most importantly, while we recognize the health system constraints relative to poor siting and design of ART centres, which exposed PLHIV to public knowledge of their status, we recommend that GHS could incorporate strong advocacy for the protection of the rights of PLHIV through the implementation of anti-HIV stigma legislation that criminalizes HIV-related stigma and discrimination (Ayiigah *et al*., 2024). While it is anticipated that these measures would deter or persuade the population to adopt accepting attitudes towards ART clients, PLHIV should be counselled and empowered to be resilient and lead productive and quality lives. These recommendations could be implemented using Ghana’s extensive decentralized health system to potentially cut costs and ensure sustainability of any gains.

## Strengths and limitations of the study

Exploring stigma and bypassing behaviours of people living with HIV in the study was best suited for qualitative study to enable a deep understanding of participants’ experiences, beliefs and perceptions concerning ART services uptake. The approach offered rich and relevant insights, which would have been missed in quantitative study. Beyond this, it offered flexibility to adapt interview questions and probe into non-verbal cues and issues during the data collection. Obtaining the expressed views of PLHIV around bypassing was important to know the individualized challenges accessing ART. However, the results should be read against these limitations. First, this study was conducted in selected health facilities in the region using convenient and purposive sampling techniques. Therefore, the findings may not be applicable to other geographical settings in Ghana, because critics question the bias around such sampling procedures. We also sampled some of the providers and PLHIV, which may not represent the full range of experiences influencing bypassing behaviours within the broader group of PLHIV. The study was facility-based, and did not allow adequate time to interact with participants. HIV and ART uptake is still a sensitive issue with the potential of causing stress. For matters of confidentiality to PLHIV especially because of the poor siting of ART Centres with the hospital facilities; we were constrained in order not to cause distress to them. However, this was the first study on bypassing primary ART facilities in the study region, and given the quality of participants, coupled with the depth of issues presented, it is reasonable to say that relevant data were generated to provide an appropriate context on fear of stigma and bypassing primary ART facilities by ART clients.

## Conclusion

Stigma is a serious challenge that stakeholders in the fight against HIV fight need to recognize in order to formulate new strategies to specifically address this issue. The study provides ample evidence of the occurrence of various forms of bypassing primary ART facilities in Ghana. We found that irrespective of its form, the main reason for its occurrence is fear of stigma and discrimination directed at ART clients in the study setting. The analysis also revealed that entrenched cultural norms and values and the population’s low awareness of the efficacy of ART are the main drivers of the processes of stigma and discrimination towards ART clients in the study setting. Addressing the eclectic nature of the drivers of stigma and discrimination requires a packaged intervention that focuses on awareness creation to undo the entrenched negative cultural beliefs about PLHIV, increase understanding of the efficacy of ART, implement existing anti-HIV stigma legislation, and empower PLHIV to be confident, adhere to ART regimens and live longer, healthier lives.

## Data Availability

The data that support the findings of this study are contained in the manuscript.
